# Supplementary material for: Effects of Environmental Hypoxia on Serum Hematological and Biochemical Parameters, Hypoxia-Inducible Factor (hif) Gene Expression and HIF Pathway in Hybrid Sturgeon (Acipenser schrenckii ♂ × Acipenser baerii ♀)
Source: Genes (Basel). 2024 Jun 5;15(6):743. doi: 10.3390/genes15060743 (PMC11203381; doi:10.3390/genes15060743)
Supplement: Supplementary file 1 [file genes-15-00743-s001.zip › genes-3002178-supplementary.pdf]

genes

MDPI

Supplementary Materials:

Table S1 Primers used for qPCR

Gene name	primers sequence (5’-3’)
hif-1α	AGGCATCTGTGGATAGTGG<br>TAACGGGTGAGCTGTGGT
hif-2α	TACTGCCCAGAGGAGCTGTT<br>CTGACTGCTCACTGCCTGAC
hif-3α	CTATCGCTTCCTGGTCAAGC<br>CACCCCACTGAGGATGAAGT
hif-1β	CCCTTGACTTCGATGACGAT<br>TGGTTCTCTCTGGCAAACCT
hif-2β	AAAAGAGGGAGAAGCACA<br>ACCGTCAACCCATTCATA
hif-3β	CCCTCACAGTATGGACAG<br>GGCGAAGACCCTCTAATC
18S	TCAACACGGGGAACCTCAC<br>GACAAATCGCTCCACCAAC

Table S2 Expression levels of function genes in HIF pathways in livers of hybrid sturgeon 24 h after hypoxia challenge

Transcript ID	log_2FoldChange	P-value	Pattern	Description
c249319_g1	1.47	1.97E-02	increased	*hif-1α*
c251157_g2	0.26	4.97E-02	increased	*hif-2α*
c243941_g2	-0.38	3.95E-02	decreased	*hif-3α*
c251232_g1	0.28	1.97E-02	increased	*hif-1β*
c245117_g1	0.55	4.84E-02	increased	*hif-2β*
c249981_g1	0.53	1.17E-02	increased	*hif-3β*
c252027_g1	1.61	2.81E-02	increased	*glut1*
c223598_g1	2.43	1.82E-08	increased	*gys*
c235875_g2	1.52	2.02E-04	increased	*ldha*
c248514_g2	2.32	6.59E-10	increased	*gatm*
c226845_g1	1.44	1.35E-04	increased	*angptl3*
c246477_g1	0.45	1.70E-02	increased	*vegf*
c243908_g1	-0.53	1.16E-02	decreased	*vhl*
c248156_g3	1.17	6.56E-03	increased	*phd2*

glut1: *glucose transporter protein type 1*; *gys*: *glycogen synthase 1*; *ldha*: *lactate dehydrogenase A*; *gatm*: *glycine amidinotransferase*; *angptl3*: *angiopoietin-related protein 3*; *vegf*: *vascular endothelial growth factor*; *vhl*: *von hippel–lindau*; *phd2*: *prolyl hydroxylase domain-containing protein 2*;
